# High Coverage Mitogenomes and Y-Chromosomal Typing Reveal Ancient Lineages in the Modern-Day Székely Population in Romania

**DOI:** 10.3390/genes14010133

**Published:** 2023-01-03

**Authors:** Noémi Borbély, Orsolya Székely, Bea Szeifert, Dániel Gerber, István Máthé, Elek Benkő, Balázs Gusztáv Mende, Balázs Egyed, Horolma Pamjav, Anna Szécsényi-Nagy

**Affiliations:** 1 Institute of Archaeogenomics, Research Centre for the Humanities, Eötvös Loránd Research Network, Tóth Kálmán Street 4, 1097 Budapest, Hungary; 2 Doctoral School of Biology, Institute of Biology, ELTE Eötvös Loránd University, Pázmány Péter sétány 1/C, 1117 Budapest, Hungary; 3 Department of Bioengineering, Socio-Human Sciences and Engineering, Faculty of Economics, Sapientia Hungarian University of Transylvania (Cluj-Napoca), Piața Libertății 1, 530104 Miercurea-Ciuc, Romania; 4 Institute of Archaeology, Research Centre for the Humanities, Eötvös Loránd Research Network, Tóth Kálmán Street 4, 1097 Budapest, Hungary; 5 Department of Genetics, Faculty of Natural Sciences, ELTE Eötvös Loránd University, Pázmány Péter sétány 1/C, 1117 Budapest, Hungary; 6 Department of Reference Sample Analysis, Institute of Forensic Genetics, Hungarian Institutes for Forensic Sciences, Mosonyi Street 9, 1087 Budapest, Hungary

**Keywords:** Székelys, Hungarians, Transylvania, mitogenome, Y-chromosome, genetic diversity, phylogeography, phylogenetics

## Abstract

Here we present 115 whole mitogenomes and 92 Y-chromosomal Short Tandem Repeat (STR) and Single Nucleotide Polymorphism (SNP) profiles from a Hungarian ethnic group, the Székelys (in Romanian: Secuii, in German: Sekler), living in southeast Transylvania (Romania). The Székelys can be traced back to the 12th century in the region, and numerous scientific theories exist as to their origin. We carefully selected sample providers that had local ancestors inhabiting small villages in the area of Odorheiu Secuiesc/Székelyudvarhely in Romania. The results of our research and the reported data signify a qualitative leap compared to previous studies since it presents the first complete mitochondrial DNA sequences and Y-chromosomal profiles of 23 STRs from the region. We evaluated the results with population genetic and phylogenetic methods in the context of the modern and ancient populations that are either geographically or historically related to the Székelys. Our results demonstrate a predominantly local uniparental make-up of the population that also indicates limited admixture with neighboring populations. Phylogenetic analyses confirmed the presumed eastern origin of certain maternal (A, C, D) and paternal (Q, R1a) lineages, and, in some cases, they could also be linked to ancient DNA data from the Migration Period (5th–9th centuries AD) and Hungarian Conquest Period (10th century AD) populations.

## 1. Introduction

The Székelys (also known as Szeklers or Seklers) are a Hungarian-speaking minority that has been living in Transylvania (Romania) for more than 800 years. Several theories have been elaborated about the origin of the Székelys over time, which is still an unresolved question to this day. They have been identified as descendants of Migration Period Hunnic, Avar, and latter-arrived Kabar, Volga Bulgarian (Onogur), and Hungarian ethnic groups. The story of their European Hunnic (5th century AD) origin was elaborated by medieval Hungarian chroniclers (who, by doing so, increased the authority of the Árpád dynasty and created the legal basis for the Hungarian conquest). Therefore, the Székelys’ own Hunnic “tradition” seems to have developed secondarily as a result of these efforts. Due to the lack of evidence, modern historiography and archaeology do not consider the Székelys to be of Hunnic origin [1]. The European Avars ruled the Carpathian Basin between the late 6th–early 9th centuries AD and also settled southern and middle Transylvania along the Mureș River. Some scholars regard the Székelys to be the remnants of the late Avar population who, according to their assumption, spoke the Hungarian language [2]. Although this question may contain some realistic elements, research still needs to explain and prove it in detail. Other scholars consider the ancestors of the Székelys as ethnic groups separated from the Volga Bulgarians, who were thus of Turkish origin [3]. According to this idea, the accession of these Bulgarian tribes to the Hungarians would have taken place even before the Hungarian conquest of the Carpathian Basin in 895 AD [3]. The theory, however, that attempted to connect the Székely folk name with the Askal/Äskäl tribe of the Bulgarians turned out to be linguistically incorrect [4]. Other experts assume that the Székelys were originally Hungarian ethnic groups who guarded the various border sections of the early Hungarian Kingdom, primarily at the western ends, and later, in the 12th–13th centuries, the majority of them were resettled in Transylvania in order to stop the Cuman and later Tatar incursions that threatened the eastern borders [5]. The first written mention of the Székelys originates from the 12th century, mentioning them as military auxiliaries of the Hungarians along the Pechenegs, still in the western border region [6,7,8].

At the moment, the research faces a serious contradiction. On the one hand, the Székely population had and has its own name and traditions, which, after observing typical military service to the king, may seem like an “auxiliary people” who joined the Hungarians, whose territorial organization was not the usual county of the rest of the Hungarians, but the district/*sedes* typical of foreign ethnic groups. The seemingly distant connections of this population towards Asia and the late Avar period have recently been also raised by physical anthropological research [2]. On the other hand, their archaeological findings from the Árpád period do not differ from the findings of the rest of the Hungarian population and do not show special oriental features, just as their Hungarian dialect does not indicate a change of language and the subsequent acquisition of the Hungarian language. Their placement in small patches at critical points of the country’s border reflects the conscious organization of the early Árpádian kings for the sake of border and land protection. Their significant medieval privileges were based on their continuous military service, which made them important actors of the time, and this continued in the early modern period in the territory of the independent Principality of Transylvania. To this day, their mother tongue is clearly spoken Hungarian [5,9,10,11].

A branch of the Hungarian ethnic group known as the Csángó is also related to the Székelys, the Csángós of Ghimeş/Gyímes, who moved from the area of Ciuc/Csík district to the valley of the Trotuş/Tatros River on the border of Transylvania and Moldavia from the early modern period (or perhaps earlier) and whose language is thus closely related to the Csík dialect [12]. Due to their close relationship with the Székelys, the Ghimeş Csángós are also analyzed in this study from published sources [13,14,15,16]. They should not be confused with other Csángó groups living in other areas of Western Moldavia and speaking a different dialect, whose origins are also different.

In recent decades, molecular genetics studies have described the genetic make-up of some of the urban Székely (Miercurea Ciuc/Csíkszereda and Corund/Korond) and Ghimeş Csángó groups, investigating maternally inherited mitochondrial DNA (mtDNA) [14,15,17] and paternal Y-chromosomal [13,16,18] lineages. Most of these studies lacked thoroughly planned and executed sample collection; thus, one cannot be sure that all sample donors had local ancestors. The studies revealed an increased number of Central or Eastern Asian lineages in the Székely population compared to other Hungarian-speaking populations. In addition to former uniparental studies, genome-wide genotype data from 24 Székely individuals from the commune of Corund were analyzed and compared with genotype data of Hungarians [19].

Besides Ghimeş Csángós and Székelys, the population of Hungary has also been investigated [20]. Hungarian paternal lineages from Hungary were reported by Völgyi et al. in 2009 and by Pamjav et al. in 2017 [21,22]. There is scarce genetic data available from the Romanian population—only one mitochondrial DNA sampling has been reported, and haplogroup results, which are based on mtDNA control region and coding marker data, were made accessible by Cocos et al. in 2017 [23].

The genetic research on the Székely population does not currently have databases containing complete mitochondrial genomes that would have been based on accurate sampling. Both of these have great importance in evaluating genetic continuity between present-day and ancient populations.

In this study, we aimed to reconstruct the uniparental gene pool of the Székely population that existed 100–150 years ago by finding elderly sample donors living in isolated villages and carefully documenting their genealogies. Furthermore, we aimed to monitor any regional genetic structure discrepancies of the Hungarian-speaking population and to confirm preliminary uniparental genetic studies that revealed an increased number of Eastern Eurasian lineages in isolated populations compared to populations of larger cities nearby. We present new genetic data containing 115 newly sequenced whole mitochondrial genomes and 92 Y-chromosomal Short Tandem Repeat (STR) haplotypes and haplogroups of a Székely population that has not been sampled before and compare them to recent Eurasian and available ancient DNA (aDNA) data to gain further knowledge about their genetic history.

## 2. Material and Methods

### 2.1. DNA Samples, Extraction, Amplification, and Sequencing

**Figure 1:**
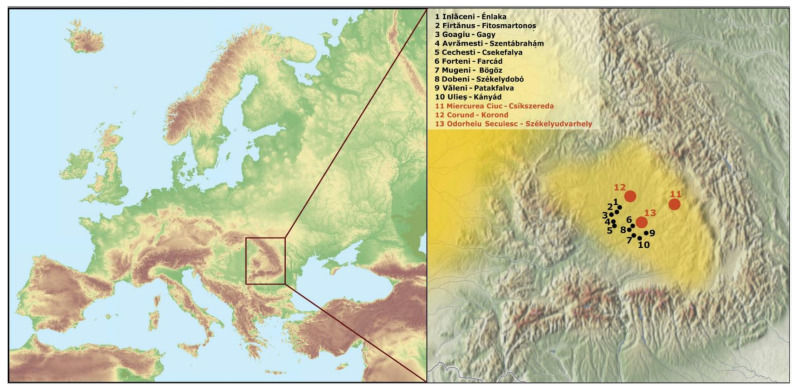
Map of Europe and the Transylvanian part of Romania showing the Székely villages where the DNA samples were collected (in black). The yellow shadings indicate the settlement areas of the Hungarian-speaking populations, including the Székelys. Red circles indicate previously collected and published Székely datasets (Egyed et al., 2007; Tömöry et al., 2007 [14,15]) and the city of Odorheiu Secuiesc. The map of Europe was downloaded from MAPSWIRE [25], licensed under CC BY 4.0, ©2022, Stefan Fischerländer. The map of the Carpathian Basin is owned by the Institute of Archaeology, Research Centre for the Humanities, Eötvös Loránd Research Network; modifications were made in Adobe Acrobat Pro DC and Inkscape 1.1.1.

Samples were collected with buccal swabs by researchers from ELRN RCH Institute of Archaeogenomics, the ELTE University of Budapest, and the Sapientia Hungarian University of Transylvania. The samples were taken from 115 (with two exceptions) unrelated individuals of the Székely population of Transylvania, Romania. The selected individuals spoke Hungarian as their mother tongue and had Hungarian surnames. All sampled individuals agreed and gave their written consent to the anonymous use of their samples in this study. Their ancestors had been documented for two generations, and these ancestors were born in the same region of Transylvania and had declared themselves as Székelys. The following villages in Harghita County were included in the sampling, near the town Odorheiu Secuiesc, which has appeared in written records from the 14th century onward [24]: Inlăceni/Énlaka, *n* = 9; Firtănuș/Firtosmartonos, *n* = 7; Ulieș/Kányád, *n* = 21; Mugeni/Bögöz, *n* = 13; Goagiu/Gagy, *n* = 11; Avrămești/Szentábrahám, *n* = 13; Cechești/Csekefalva, *n* = 9; Dobeni/Székelydobó, *n* = 12; Văleni/Patakfalva, *n* = 7; Forțeni/Farcád *n* = 13 (Figure 1, Supplementary Table S1).

DNA was extracted with QIAamp DNA Mini Kit (Qiagen) according to the producer’s buccal swab spin protocol. The concentration of the samples was measured with Qubit^TM^ dsDNA High Sensitivity Assay Kit (Thermo Fisher Scientific, Waltham, MA, USA).

The amplification of the whole mtDNA was performed with the Expand^TM^ Long Range dNTPack kit (Sigma Aldrich) according to Fendt et al., 2009 [26] (primer sequence 5′–3′, forward ‘A’ (FA): AAATCTTACCCCGCCTGTTT; reverse ‘A’ (RA): AATTAGGCTGTGGGTGGTTG; forward ‘B’ (FB): GCCATACTAGTCTTTGCCGC; reverse ‘B’ (RB): GGCAGGTCAATTTCACTGGT). We amplified the mtDNA in two fragments and modified the PCR program according to the length of the fragments. Conditions used for long-range PCR consisted of an initial denaturation step of 2 min at 92 °C followed by 10 cycles of denaturation at 92 °C for 10 s, annealing at 60 °C for 15 s, and elongation at 68 °C for 8 m 30 s, 10 cycles of denaturation at 92 °C for 10 s, annealing at 60 °C for 15 s, and elongation at 68 °C for 8 m 50 s, 15 cycles of denaturation at 92 °C for 10 s, annealing at 60 °C for 15 s, and elongation at 68 °C for 9 m 10 s, and a final elongation step at 68 °C for 7 min. The amplification reaction was checked on 0.8% agarose gel and visualized after EcoSafe staining with UV transillumination. We pooled the two separately amplified fragments, then purified the amplicons with the QIAquick PCR Purification Kit (Qiagen). The concentration of the PCR products was measured with Qubit^TM^ dsDNA Broad Range Assay Kit (Thermo Fisher Scientific).

NEBNext Ultra II FS DNA Library Prep Kit was used for the preparation of the mtDNA libraries. The products were checked with the Agilent D1000 ScreenTape Assay on the 4200 Tapestation system. Next-generation sequencing was performed on the Illumina Miseq System (Illumina) using Illumina Miseq Reagent Kit V2 (2 × 150 cycles) sequencing kit. Indexed libraries’ final concentrations were adjusted to 4 nM. Samples were pooled together, taking into account the calculated coverage to be achieved. Five percent of PhiX was used to increase the heterogeneity of samples.

We analyzed 92 male samples in the Laboratory of Reference Sample Analysis of the Department of Genetics, Directorate of Forensic Expertise, Hungarian Institute for Forensic Sciences in Budapest. DNA was surveyed for STR variation using the Promega PowerPlex Y23 for the Székely population, including 23 Y-STR loci. ABI3130 Genetic Analyzer and GeneMapper ID-X v.1.2 software was used for fragment analyses of PCR products. The results of the Y-chromosomal STR analyses were verified by haplogroup-defining Single Nucleotide Polymorphism (SNP) markers (see Supplementary Table S2) on ABI 7500 Real-time PCR instrument using SDS.1.2.3 software.

### 2.2. Pre-Processing of the Sequencing Data

A custom in-house bioinformatic pipeline was applied to the Illumina sequencing data [27]. Paired-end reads were merged together with the SeqPrep master [28]. At a maximum of one mismatch, the one base with higher base quality was accepted, and the overlapping reads with two or more mismatches were discarded. The pre-processed reads were mapped to the rCRS reference sequence using BWA v.0.7.5 [29] with a MAPQ of 30. The majority rule was applied for the consensus sequence calling for the high-coverage mitogenomes. No indels were examined in the process. Samtools v.1.3.1 [30] was used for further data processing, such as indexing, removing PCR duplications, and creating bcf files.

Mitochondrial haplogroup determinations were performed by HaploGrep2 [31], which uses Phylotree mtDNA tree Build 17 [32,33]. We analyzed heteroplasmy using Mutserve [34], which can detect heteroplasmy of at least 1% (Supplementary Table S10).

Y-haplogroups were assigned based on Y-STR data using nevgen.org, as well as based on Y-SNP genotyping by TaqMan assay on a Real-time PCR platform. Terminal Y-SNPs were verified on the Y tree of ISOGG version 15.34 [35].

We created and visualized the median-joining (MJ) network of the whole mitochondrial genomes of our dataset with the PopArt program [36]. The input file of PopArt was made by DnaSP [37].

### 2.3. Phylogenetic Analysis of the mtDNA

For neighbor-joining mtDNA phylogenetic trees, we collected all publicly available mtDNA sequences from databases (most of the data used are from the NCBI database; IDs and sources of other data are available in Supplementary Table S6), then, we kept the sequences that belonged to the same or similar haplotype as our samples. Subsequently, we divided this filtered dataset into larger groups based on haplogroups consisting of 50–150 sequences each. We aligned sequences in each group with ClustalO within SeaView [38]. The alignments were checked and corrected manually where necessary. Comparing to the rCRS sequence, we deleted the following positions: bases 42, 57, 291–317, 447–458, 511–524, 568–573, 594–597, 1718, 2217–2226, 3106–3110, 3159–3167, 5890–5894, 8272–8281, 16184–16193. Next, neighbor-joining (NJ) trees were generated by PHYLIP version 3.6. [39]. The phylogenetic trees were then drawn using Figtree version 1.4.2. [40].

### 2.4. Population Genetic Analysis

Principal component analysis (PCA) was performed based on the mtDNA haplogroup frequencies of 56 modern and two ancient populations (see the list of populations in Supplementary Table S4). In the PCA of the modern populations, we considered 36 mitochondrial haplogroups. The PCAs were carried out using the prcomp function in R v4.0.0. [41] and visualized in two-dimensional plots with two principal components (PC1 and PC2 or PC1 and PC3).

For a Ward-type hierarchical clustering, we involved the same population datasets as for PCAs. Based on the mtDNA haplogroup frequencies, we calculated PC-scores in R v4.0.0 [41], then applied PC1–PC6 scores using the Euclidean distance measurement and ward.D method. We visualized the results as a dendrogram with the hclust library.

To calculate the inter-population variability of the mtDNA genetic profiles characteristic of the three Székely populations, we performed an analysis of molecular variance (AMOVA) using Arlequin v3.5.2.2 software [42].

We calculated population pairwise F_ST_ and linearized Slatkin F_ST_ values based on the whole mitochondrial genome sequences of 3981 modern-day individuals (classified into 21 groups) and 362 ancient individuals (classified into 7 groups) using Arlequin v3.5.2.2. [42] with the following settings: Tamura & Nei substitution model with 10,000 permutations, a significance level of 0.05, and a γ value of 0.3.

We used the same linearized Slatkin F_ST_ values for clustering in Python using the seaborn [43] clustermap function (parameters: metric = ‘correlation’, method = ‘complete’).

### 2.5. Analysis of Y-STR Variations

MJ networks were constructed using Network v10.1.0.0, and the results were visualized with Network Publisher v2.1.2.5 [44]. The following settings were used in Network v10.1.0.0: network calculation: median-joining [45], optional post-processing: maximum parsimony calculation [46] (selected option: network containing all shortest trees, and list of some of the shortest trees sufficient to generate the network) and in Network Publisher, shortest tree visualization was applied and colored according to the haplogroups and sample provenance.

## 3. Results and Discussion

The sample pool of this study consisted of 92 male and 23 female participants, all Székely individuals from the Transylvanian part of Romania (for detailed information, see Supplementary Table S1). We performed whole mitochondrial genome enrichment and next-generation sequencing (NGS) to obtain 115 complete mitogenomes. In addition, we investigated the Y-STR profiles (23 STRs) and Y-SNP data of the 92 male individuals (see Supplementary Table S2).

### 3.1. Maternal Lineages in the Dataset

#### 3.1.1. Haplogroup-Based Analyses

One hundred and fifteen high-coverage mitochondrial genomes were obtained with NGS methods (from 111.46× to 276.83× coverage), with a mean coverage of 233.56×. The 115 complete mitochondrial genomes were classified into 72 different haplotypes. These mitochondrial haplotypes are mainly present in European regions, but there were several haplotypes predominantly found in present-day Asian or Near Eastern populations. The new dataset consisted of the following macrohaplogroups: A, C, D, H, HV, HV0, I, J, K, T, U, N, R, V, W, and X. A list of the mtDNA subhaplogroups found in the Székely population is in Supplementary Table S3.

**Figure 2:**
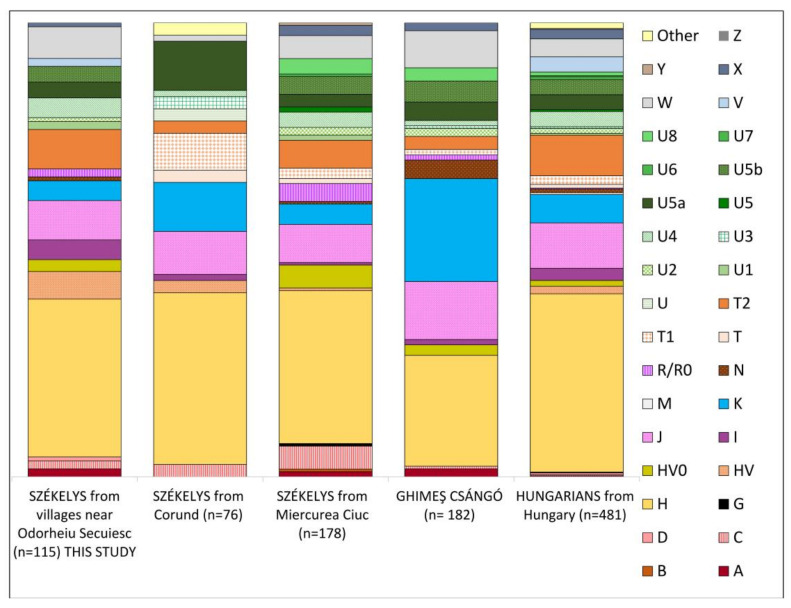
Mitochondrial haplogroup composition of the investigated Székely population around Odorheiu Secuiesc compared to other Székely populations [15,17], the Ghimeş Csángó population [17], and Hungarians living in Hungary (see references in Supplementary Table S4).

The overall mitochondrial haplogroup composition of the investigated Székely population was similar to the formerly described Székely populations in Miercurea Ciuc and Corund and to the Hungarian population in Hungary as well [15,17]. Around Odorheiu Secuiesc, most of the individuals belonged to haplogroup H (34.8%) —as expected in a European population [47], and as had been observed in the case of earlier studied Székely (37.8%), Hungarian (39.3%), and Ghimeş Csángó (24.4%) populations (see Figure 2). Compared to the populations of Miercurea Ciuc and Corund, some differences were conspicuous. We observed a higher proportion of haplogroups I (4.3%), T2 (8.7%), HV (6.1%), and W (7%), and a lower proportion of haplogroup K (4.3%) than in previous studies; furthermore, no T1 was present in our dataset. All three Székely populations had a significant proportion of mitochondrial haplogroups with Eastern Eurasian prevalence (A, B, C, D, G, and Y). Their proportion was higher in the Székely population of Miercurea Ciuc (7.86%) than around Odorheiu Secuiesc (4.35%) and in Corund (2.7%). The Ghimeş Csángó population stood out slightly in the comparison due to its higher proportion of the haplogroup K (22.7%) and lower proportion of H (24.4%) [17].

**Figure 3:**
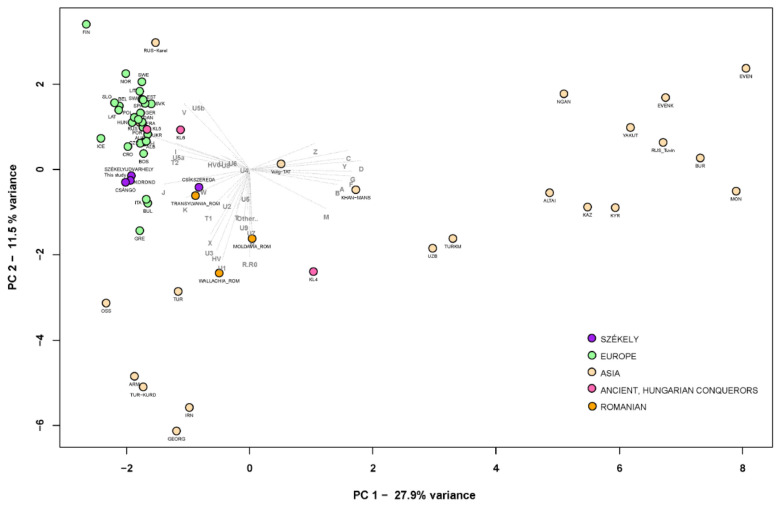
PCA plot with 56 modern and three ancient populations (36,803 samples), representing first and second principal components (39.4% of the total variance): PCA analysis based on mtDNA haplogroup frequencies in Eurasian modern populations and three ancient populations. The selected ancient populations are the Hungarian Conquest Period (10th century AD) populations of the Carpathian Basin [48,49,50,51]. KL4-5-6 groups indicate different cemetery types in the Hungarian Conquest Period, as used in Szeifert et al., 2022 [52]. The investigated Székely population and previously examined Székely groups are marked in purple, the ancient populations from Hungary in pink, modern-day Romanian populations in orange, other modern-day Europeans in green, and Asian populations in beige. The PCA shows a clear separation of Eastern (right side of the plot) and Western (left side of the plot) populations. For further information, see Supplementary Table S4.

We used PCA in order to visualize the population genetic relatedness based on mtDNA profiles and frequencies of the different populations (see Supplementary Table S4). The investigated Székely population (Odorheiu Secuiesc) was positioned among European populations, closest to other Székelys, Ghimeş Csángós, Croatians, Bosnians, modern Czech populations and Transylvanian Romanians (see Figure 3). It was not possible to further examine all connections at the complete mitogenome sequence level due to the lack of whole mitogenome data in some populations.

Data on the PCA were also displayed using the hierarchical ward-clustering method (see Supplementary Information Figure S1). The clustering confirms the connection of the studied Székely group with Europeans but also separates the Ghimeş Csángó and Corund groups from the group around Odorheiu Secuiesc. This difference in the PC1-2 plot is also visible on the PC3, where the Odorheiu Secuiesc group becomes distant from the others (Supplementary Information Figure S2).

Since the other two Székely groups were only analyzed for Hypervariable Region I of the mitochondrial genome, the sequence-based comparison of the three groups (with limited conclusions) is discussed in the Supplementary Information.

#### 3.1.2. Whole Mitogenome Sequence-Based Evaluations

##### F_ST_ Analyses

**Figure 4:**
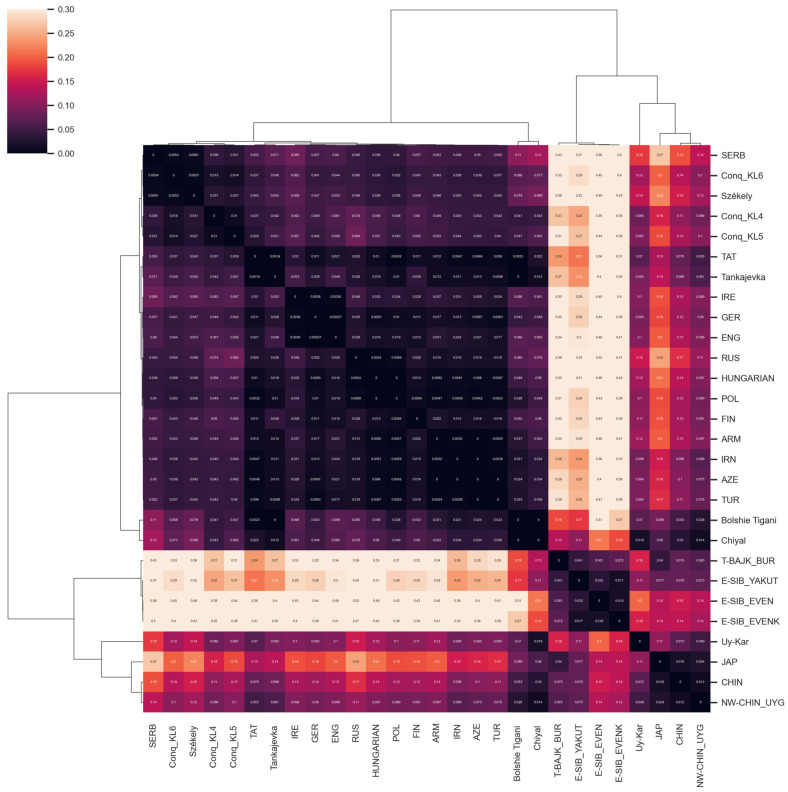
Heatmap of pairwise F_ST_ values (based on whole mitogenome sequences) for the modern Székely group and 27 reference populations with a color scale ranging from yellow to dark purple. The lighter block colors indicate larger genetic differentiation, whereas the darker colors show closer genetic affinities between the pairs of populations. The European groups all show great similarities with each other. The Székelys cluster on the European branch with Serbians and Conquest Period Hungarian groups (KL4-5-6 group description is defined in Figure 3 caption); in addition to these, the Hungarian and Polish groups show the closest links. We calculated the clustermap in Python using the seaborn clustermap function with parameters: metric = ‘correlation’, method = ‘complete’.

We analyzed the whole mitogenomes (16,569 base pairs) at the DNA sequence level and calculated Slatkin F_ST_ values (see Supplementary Table S5). A heatmap with clustering of F_ST_ values was created to visualize the genetic differentiation of the examined populations (Figure 4). The Székelys cluster on the European branch with Hungarians, where the Serbians and the Conquest Period Hungarians are the most similar to them. Whole mitochondrial data are missing from Romania, and the Slovakian and Czech datasets are also limited; therefore, the resolution of that analysis is restricted. Among the ancient populations, the KL6 group, which was discussed by Szeifert et al. as comprising large village cemeteries opened in the 10th century and used until the 11th and 12th centuries in the Hungarian Kingdom [52], was the closest to the Székelys.

##### Phylogenetic Analyses of the Székely Maternal Lineages

**Figure 5:**
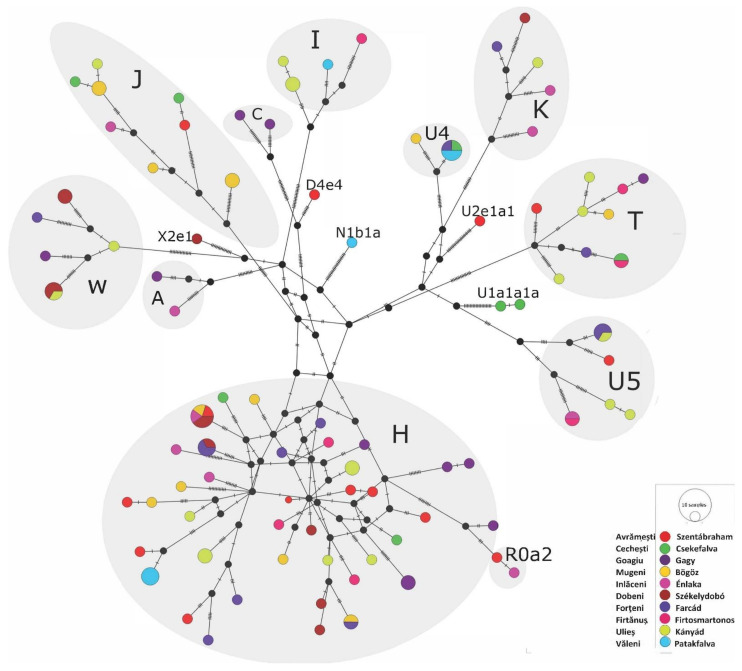
Median-joining network of 115 modern-day Székely mitogenomes. The sequences contained 484 variable sites and belonged to 72 haplotypes. The figure was created with the PopArt program.

The median-joining network of the Székely mitogenomes showed the variable distribution of the maternal lineages among the sampled villages (see Figure 5). Most of the haplogroups were shared among the villages, and shared lineages were also found among certain H, T, U4, U5, and W haplotypes. Three individuals belonging to the described Eastern Eurasian haplogroups (A+152+16362, C4a1a3, C5c1a) originated from the same village (Goagiu), although we detected Eastern Eurasian haplogroup types in the Avrǎmeşti (D4e4) and Inlǎceni (A12a) villages as well.

Since analyses of mitogenomes in pools did not lead to differentiation of distant present-day European populations, we investigated individual maternal lineages in the following in order to monitor the connections of the Székely maternal lineages to early Hungarian populations, among others.

**Figure 6:**
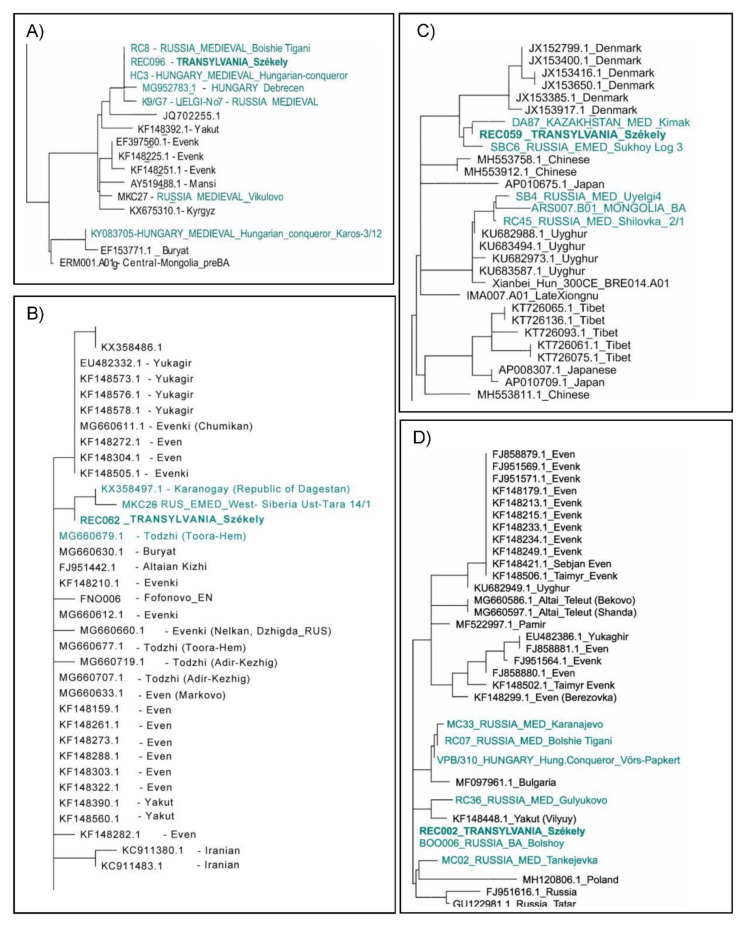
Parts of neighbor-joining phylogenetic trees of mitochondrial haplogroups. (**A**) Mitochondrial haplogroup A12a, (**B**) C4a1a3, (**C**) A+152+16362, and (**D**) D4e4. Samples highlighted in turquoise are historically relevant to the Székely samples. Most of the data used for the neighbor-joining mitochondrial phylogenetic trees are from the NCBI database; IDs and sources of other data are available in Supplementary Table S6.

On the A12a phylogenetic tree (Figure 6A), a modern-day Hungarian sample and a Hungarian sample from the time of the Hungarian conquest (10th century, Harta_HC3), as well as two samples from the 9th–10th-century Volga-Ural region (Bolshie Tigani RC8 and Uyelgi-No7, [49,52]), cluster together with the examined modern-day Székely sample. The Conquest Period and the Bolshie Tigani individuals had identical mitochondrial DNA sequences to the Székely individual. Based on this tree, we assume that the phylogenetic lineage A12a came from the Volga region and was also present at the time of the Hungarian conquest (late 9th–10th century). The newly reported samples within the A12a subgroup caused some changes in the nomenclature within the A12a tree that we present in Supplementary Information Figure S3. The Székely sample described here has been ordered to a new subgroup named A12a2b.

On the partial C4a1a3 neighbor-joining phylogenetic tree (Figure 6B), the MKC26 refers to a sample that originated from the 6th–8th-century West-Siberian Ust-Tara archaeological site of the Nizhneobskaya culture [52]. The population of this culture was probably proto-Ob-Ugric (Southern-Khanty), although it showed typical Hun-period cultural traits [53]. The other sample that shares a branch with the Székely sample originated from the Karanogay ethnicity (Turkic ethnic group), Dagestan, and the adjacent ‘Todzhi’ sample was also from a Turkic ethnic group, a group of Tuvans. This tree represents the mixed nature of the C4a1a3 lineage, which despite its prevalence in Turkic-speaking ethnic groups, may also have originated from Western Siberia in the Székely gene pool. Nevertheless, we do not have immediate proof of that hypothesis in the form of linking lineages from the Volga-Ural region, where the ancestors of Hungarians settled until the 9th century.

On the A+152+16362 tree (Figure 6C), a sample from Cis-Uralic Sukhoy Log cemetery (7th–8th centuries) [52], as well with the latter contemporaneous, as ‘Kimak’-reported individual from the Central Steppe [54], can be found in close proximity to the Székely sample. The relationships between samples from the Volga region Early Medieval sites Karanayevo, Bolshie Tigani, Gulyukovo, Tankeevka, from the Conquest Period Transdanubia and the Székely sample on the D4e4 phylogenetic tree (Figure 6D) suggest a possible connection between these sublineages via the conquerors. However, the Székely lineage has a basal character and is identical to a lineage detected in the Bronze Age of Bolshoy Oleny island in Kola Bay. It is, therefore, possible that D4e4 is an originally Northeastern European maternal lineage that reached the Carpathian Basin via a different migration.

### 3.2. Paternal Lineages in the New Székely Dataset

The population genetic investigation of non-recombining Y-chromosomal markers like Y-STRs and Y-SNPs can be used to trace back paternal lineages in time and describe phylogeographic structures and diversities of populations.

#### 3.2.1. Haplogroup-Based Analyses

Y haplotypes from 23 Y-STR markers were obtained from 92 men out of the 115 individuals sampled. The haplogroup predictions were confirmed by selective Y-SNP typing (see methods and Supplementary Table S2). In this dataset, we found eight Y macrohaplogroups (E, G, H, I, J, Q, R, and T), which included 21 different subhaplogroups based on SNP typing. Some of these Y subhaplogroups are predominantly found among Inner Asian (R1a-Z93) populations, and South Asian/European Roma (H-M52), as well as Northern Eurasian (Q-M242) people (in a total of seven samples, 7.6% of all samples), the other 18 subhaplotypes are mostly referred to as European-derived types (Supplementary Table S7).

Although some studies on the Székely male populations have been published previously, their comparability with our dataset is rather limited. In 2005, Egyed et al. studied 257 Székely individuals from Miercurea Ciuc, including 89 males, typed for 12 Y-STR haplotype loci. In Csányi’s study from 2008, 13 Y haplogroups were determined in the Székely population from Corund [16]. The Y haplogroup diversity was 0.9157 in the latter Székely population, 0.9011 in the Székelys living in Miercurea Ciuc [17], and 0.8636 among the Hungarian male population in Hungary [21]. The Y-STR haplotype diversity in our studied Székely population was 0.9995, and the proportion of unique haplotypes was 97.8% using the PowerPlex Y23 System. Based on an investigation of 72 European populations comprising a total of 12,000 samples, the average haplotype diversity was higher than in the Székelys (Hd = 0.999992) using the same Y23 System [55]. The haplotype diversity of maternal lineages is comparably high, equals to 0.9941.

In 2015, Bíró et al. studied Székely haplogroups from Miercurea Ciuc (haplotypes published by Egyed et al.) [13,18] that had proven Central and Inner Asian genetic contributions (J2*-M172 (xM47, M67, M12), J2-L24, R1a-Z93, Q-M242, and E-M78 haplogroups). In their dataset, the possible maximum Central/Inner Asian admixture among the Székely male population was 7.4% [13,18]. In our results, this proportion was a comparable 7.6% in the population around Odorheiu Secuiesc. According to Bíró’s study of contemporary Hungarians from Hungary, this Central/Inner Asian admixture was estimated as only 5.1% and 6.3% in the Ghimeş Csángó male population. Examining the Bodrogköz area Hungarian dataset, these Asian-derived haplogroups appeared in 6.9% of the population [22]. Bodrogköz is a geographical area in the Upper-Tisza region in north-eastern Hungary bordered by the Bodrog and Tisza rivers. Due to its isolated nature, the dataset from Bodrogköz has been treated separately from the Hungarian data in our study. The authors assumed that its present-day population is likely to preserve ancient markers and lineages, as its former inhabitants had a better chance of surviving both Mongol and Ottoman invasions than groups living in some of the other affected regions [22].

**Figure 7:**
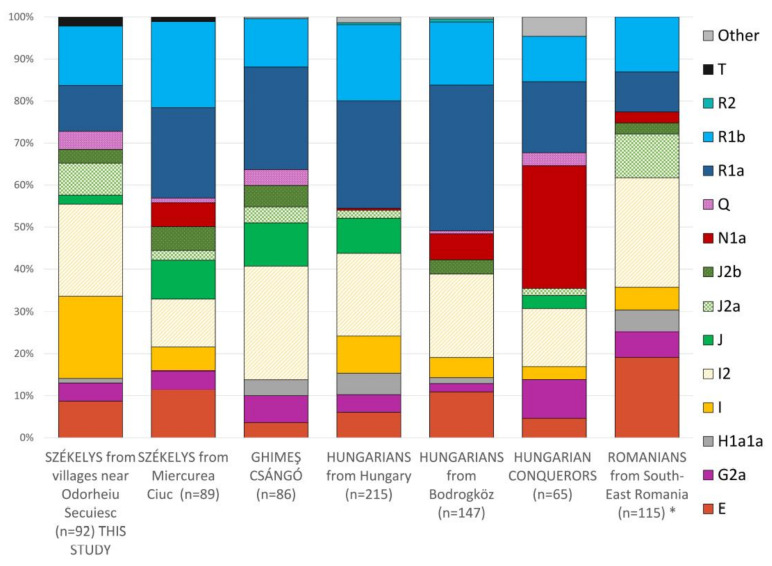
Diagram of the Y haplogroups in Székely [17], Ghimeş Csángó [17], Hungarian [21], Hungarian Conqueror [56,57], and Romanian [58] populations. * Haplogroups from the Romanian population were predicted from 17 STR data using nevgen.org.

The comparative analyses of the Y haplogroups with other Székely populations showed some level of diversity among the Székely groups. However, while N1a occurred among the Székelys in Miercurea Ciuc, the population of the Odorheiu Secuiesc region did not yield such a signal in our analysis but resulted in higher proportions of haplogroups Q (4.3%), I (19.6%) and I2a (21.8%) and lower J (2.2%) and R1a (10.9%) than in the previously studied Székelys (Figure 7).

The haplogroup R1a comprised a substantially higher proportion of the haplogroups among the Hungarian populations living in Hungary (fourth and fifth columns in Figure 7) than among either the Székely population of the present study or the Romanian population. In the Székely population we studied, R1a and R1b comprised only 25% of all haplogroups, while in the case of Hungarians in Hungary, they accounted for 45–50%. However, the ratio of I (19.6%) and J2 (10.8%) haplogroups was higher in the studied Székelys than in the Hungarians (I 13.8%, J2 1.9%). Furthermore, the frequency of haplogroup J was higher in the Székely population of Miercurea Ciuc (9.1%) than in Odorheiu Secuiesc (2.2%).

Székelys in Miercurea Ciuc showed roughly similar frequencies of G2a and E haplogroups to Székelys around Odorheiu Secuiesc; furthermore, the T haplogroup did not appear elsewhere in the comparison except in the two Székely groups. T-M70 seems to have originated from the Fertile Crescent and possibly arrived in Europe in the Neolithic with the first farmers [59,60]; today, it shows the highest frequency in East Africa and the Middle East. The Székely T-M70 samples belonged to the T1a2b1 subhaplogroup, which is rarely detected in ancient data. However, it was found in the Hungarian Conquest Period horizon of the Western Hungarian Vörs-Papkert cemetery [61,62].

The I2a1a (I2a-P37) haplotype occurred in the highest number in the studied Székelys, comprising 20% of the total haplotypes. In Hungary, 16.74% of men carry this haplotype [21]. I2a-P37 and subgroups occur at high frequencies in Sardinia (38.9% [63]) and are also present at high frequency among Balkan populations [64]. The proportion of the I2a1a Y type in the Romanian population is 17.7% [64]. The I2a-P37 group has a long demographic history in Europe. It has been suggested that this subgroup also (similarly to M253) expanded from the Southeastern European glacial refuge area after the LGM [64].

Another dominant haplogroup in our dataset is I1a1b1 (I1-L22), which is most frequent in Sweden and Finland, and represents a fairly large Nordic branch of I1. It was dispersed by the Vikings and nowadays can be frequently found in the Baltics, Britain, Poland, and Russia [65].

**Figure 8:**
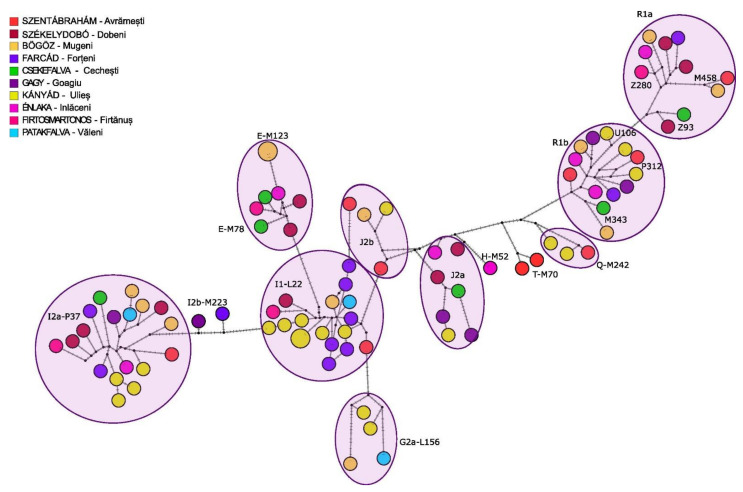
Y-network based on 23 STR data from the modern Székely population (*n* = 90). The colors indicate the villages where the sample providers live at the time of sampling. Two samples (REC105 and REC112) were excluded from the analyses due to missing or uncertain positions. Haplotypes grouped corresponding to haplogroups indicated on the network.

The distribution of Y haplogroups between the villages did not show a characteristic pattern or patrilineal system; the observed haplogroups were mixed within the region, as demonstrated in Figure 8.

#### 3.2.2. Y Chromosome Phylogenetic Analyses

In the following, we present the detailed, Y-STR-based analyses we performed on selected Y subgroups.

**Figure 9:**
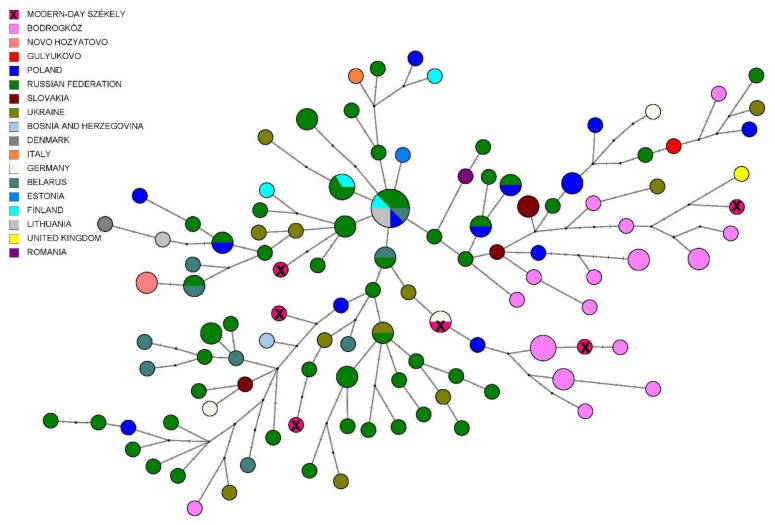
R1a-Z280 median-joining network. The analysis was performed based on 15 STRs. No larger clusters are seen in the Figure, but a Northeast European founder cluster is observable.

Our dataset comprised six samples classified into the Y chromosome R1a1a1b1a2 (R1a-Z280) haplogroup, which we visualized on a median-joining network (Figure 9). For comparison, we collected Y-STR data from the Family Tree Y-DNA database R1a page, and we filtered the samples for Y4459 SNP based on nevgen.org prediction. In addition, we included data with the same haplogroup classification from Hungary, Bodrogköz [22], and the early medieval Volga-Ural region (Novo Hozyatovo, Gulyukovo—Chiyalik culture), which may have been a settlement area of Hungarians who remained in the East after the Westward migration of the other Hungarian tribes [52,66]. On the network, individuals from the Bodrogköz region, Russia, Germany, and Poland can be found near the Székelys.

We present further median-joining networks in the Supplementary Information Figures S4–S7 for haplogroups G2a, Q1 (Q-M242), R1a1a1b1a1a (R1a-M458), and R1a1a1b2 (R1a-Z93).

Our dataset contained four samples that belonged to the Y-chromosomal haplogroup G2a, which we analyzed in further detail. In the absence of comparative data covering 23 STR, the MJ network in Supplementary Information Figure S4 is based on 17 STR. Two of the Székely G2a Y chromosomes clustered with the M406 subgroup of G2a, with individuals sampled in Tyrol, Austria [67]. This terminal SNP defines the Y-chromosomal subgroup G2a2b1 (ISOGG 2020 v15.73) [35], whereas some of the Székely samples most probably belong to G2a2b2a1a1b based on the L497 marker (ISOGG 2020 v15.73). The G-L497 subhaplogroup likely originated from Central Europe and has been mostly prevalent in European populations since the Neolithic period [60,68]. The G-L497 lineage could potentially be associated with the *Linearbandkeramik* (LBK) culture of Central Europe. The G-M406 sub-cluster is most concentrated in Cappadocia and Anatolia in Turkey nowadays [69] and has been present in that area since the early Neolithic [70,71].

The median-joining tree of Q-M242 (Supplementary Information Figure S5) placed the present Székely samples among the Bukovina-Székelys, whereas the two R1a networks did not show geographically relevant patterns. However, the R1a-M458 median joining tree (Supplementary Information Figure S6) shows the divergence of the R1a-M458 types within the Hungarian Bodrogköz population and the connection of the Székely lineages to some parts of it.

Out of four Y-chromosomal macrohaplogroup Q, three belonged to the Q1a-F1096—probably to subhaplogroups Q1a2-M25 and Q1a2a1-L715—and one belonged to the subgroup Q1b1a3-L330. These subgroups are interesting from our perspective, due to their Central Asian origin (Supplementary Information Figure S5). Q1a2-M25 is known from the second half of the 5th century AD near Sângeorgiu de Mureş, Romania [72]. Based on the discovered grave goods of the buried man at this site, and his Asian cranial features as well as artificial cranial deformation, strong Hun period traditions have been pointed out [72]. The Q1a2-M25 lineage was also demonstrably present in the Carpathian Basin in the first half of the 7th century AD from a richly furnished, high-status Avar horseman warrior’s grave in the Transztisza region, belonging to subhaplogroup Q1a2-M25 [73,74]. Ancient individuals with the same Y-chromosomal haplogroup are known from the Early Middle Bronze Age Okunevo and from the Baikal Early Bronze Age (Shamanka and Ust’Ida sites), and the Tian Shan Hunnic [54] and Hungarian Sarmatian cultural context [74] as well.

The Q1b1a3-L330 subhaplogroup was also present from the middle third of the 7th century AD in the Carpathian Basin; it was identified from a richly furnished early Avar grave [73,74], and according to a median-joining network (Figure S5 in [73]), this male had a probable Altaian or South Siberian (Tuvinian) paternal genetic origin. The Q1b1a3-L330 lineage was also present in the Proto-Ob-Ugric group (Ust’-Ishim culture—Ivanov Mis and Panovo sites), which corresponds to its Altai or Siberian origin [52,75]. The genetic imprint of the Avars in the Székely population can have multiple origins, as their 8th-century settlements were scattered throughout the central Carpathian Basin and along the Maros River in Transylvania, and part of them probably persisted after the Avar–Frankish wars as well [1].

On the R1a-Z93 median-joining tree Hungarian King Béla III and other skeletal remains originating from the Royal Basilica of Székesfehérvár [76,77] show a great genetic distance from the Székely samples, just like the Bashkirian Mari males (Supplementary Information Figure S7).

**Figure 10:**
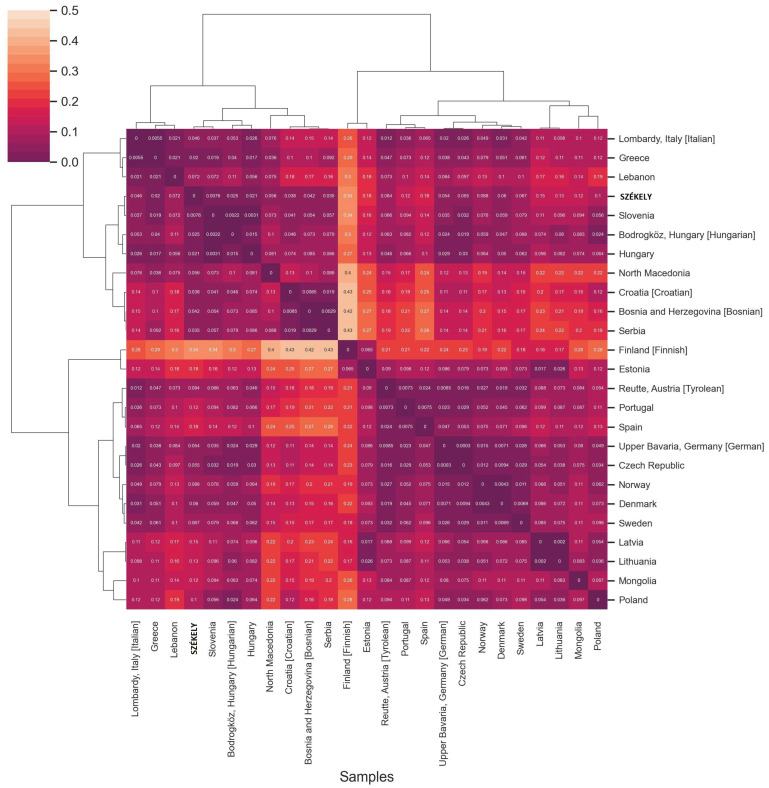
Heatmap of pairwise R_ST_ values with clustering applied for the modern Székely group and populations from Europe (color scale ranging from yellow to dark purple). We calculated it in Python using the seaborn clustermap function with parameters ‘correlation’ distance metric and ‘complete linkage’ method [43].

Examining the R_ST_ values based on Y-STR profiles (see Supplementary Table S8, Supplementary Information Figure S8), the closest population to the Székelys was the Slovenian group, with non-significant R_ST_ *p*-value; the populations with significant R_ST_ *p*-values were the Greeks, Hungarians, Hungarians from Bodrogköz, Serbians, and Croatians (Figure 10). All these reflected a strongly Southeastern European-funded base population of the Székelys with a limited proportion of surviving eastern elements.

Presumably, the signs of Glagolitic and Cyrillic origin included in their own old script are also connected with the Eastern European and Balkan connections of the Székelys. This alphabet was probably developed in the Carpathian Basin during the 10th century—using certain signs of the Turkish runic script too—and became suitable for writing short texts in Hungarian. It was used exclusively by the Székelys [78].

#### 3.2.3. Comparison of Paternal Lineages with Ancient Data

Most of the paternal lineages of the early medieval sites in the Western-Siberian and Volga-Kama regions—which regions are linked to Hungarian ethnogenesis—belong to N1a1-M46, Q1b-M346-L330, G2a2b-M406, R1a-Z93, and J2a1-Z6046 [52]. According to the publications of Fóthi et al. [57], Neparáczki et al. [56], and Csányi et al. [16], the N1a1-M46, R1b-U106, and I2a-M170 lineages were the most widespread among the conquering Hungarians they examined. This suggests that the conquerors were of diverse origins, and while the N1a1-M46 subtype originated from the Ural region, the R1b-U106 (R1b1a1b1a1a1) lineage is known from the Late Copper Age/Early Bronze Age transition in Europe [79] and is most prevalent in Germanic-speaking people nowadays [80]. These observations fit the genomic data published by Maróti et al. [62], where haplogroups N1a1a1a1a2a1c (Y13850), N1a1a1a1a4 (M2128), I2a1a2b1a1a (YP189), and R1a1a1b2a (Z94) have been presented in notable frequencies.

According to all previous studies, the N1a1 line is the most characteristic of the Hungarian conquerors—almost 30% of the lineages belong to the N1a group and also appear with lower frequencies (4–6%) in the Bodrogköz and Miercurea Ciuc populations; however, this line is completely missing from our new Odorheiu Secuiesc region dataset. The second most widespread haplogroup among the Hungarian conquerors is the R1a-M198 (16.9% of all haplogroups) which is quite common nowadays in the Odorheiu Secuiesc region (10.9%). R1b and I2 haplogroups are present in a relatively higher proportion both in the conqueror (10.8% and 13.8%, respectively) and Székely groups (14.1% and 21.7%, respectively). Most of the other haplogroups listed above—like R1b-U106, G2a, and Q1b- M346-L330—can also be found among the Székelys at a maximum frequency of 5%. The direct comparison of the data is limited, however, by the numerous allelic dropouts in the ancient STR analysis [57] and by the different levels of haplogroup resolutions obtained. 

## 4. Conclusions

In this study, we presented the maternal and paternal genetic composition of a Székely group, a Hungarian-speaking minority living around the city of Odorheiu Secuiesc in Transylvania (Romania). We carefully selected 115 sample providers with local ancestors inhabiting small villages in the area. Altogether, 115 complete, high-coverage mitochondrial genomes were produced with next-generation sequencing methods, which revealed 89 unique haplotypes that could be classified into 72 different subhaplogroups. These mitochondrial haplogroups are mainly present in European regions, but there are also some Asian- and Near Eastern-derived lineages, like A+152+16362, A12a, C4a1a3, C5c1a, and D4e4.

In this new Székely dataset, the discovery of an Asian maternal lineage (A12a) completely identical to that found in a male with typical Hungarian conqueror artifacts from the 10th-century cemetery in Harta, Hungary [81] and in the early Hungarian cemetery of Bolshie Tigani in the Volga-region, is a robust sign that some lineages in the Székely population are shared with Hungarian conquerors and are thus most probably of common origin.

The 92 paternal lineages investigated in the dataset were mainly composed of European haplogroups, but some lineages (I1-L22, T-M70, J2a-M67) stood out or showed a different distribution in their proportions than in other surrounding Székely ethnic groups. The performed Y-STR networks allowed detailed observations on paternal lineages G2a-L156, R1a-Z280, Q-M242, R1a-M458, and R1a-Z93.

We detected a strongly Southeastern European base population of the Székelys. The genetic proximity of Balkan populations may also be a consequence of the formerly inhabited areas of the Székelys in southern Hungary.

The Hunnic origin of the Székelys remains questionable in the light of the present genetic data because scarce genetic data is available from the 5th century [56,74]. Furthermore, the population living in the Carpathian Basin during the Hunnic period and in the Avar period (late 6th–9th centuries) shows large heterogeneity [74]. Here we demonstrated connections of the Székelys to the 5th–7th centuries’ population through Y-chromosomal Q Asian lineages, which, however, could have arrived repeatedly in the region in numerous epochs. The possible separation of the different immigrant waves requires larger comparative databases from the early medieval and medieval periods.

We found large among-village heterogeneity in both the maternal and paternal gene pools. Among both maternal and paternal lineages, mainly European types have been identified in comparable proportions, but in both cases, certain eastern lines can be characterized. The current Székely dataset completes the previous studies and is broadly in line with their observations. The genetic connections between the Székelys and Hungarians could be detected based on our uniparental genetic data using allele frequency analyses, in line with genome-wide haplotype data from the Corund city and other Hungarian populations [19].

Compared to previous uniparental studies involving Székelys [13,14,15,16,17,18], what is different and novel in our research is the sampling method, the selection of the participants, and the careful documentation of the ancestors, who mostly lived in the micro-region for up to two generations (Supplementary Information Figure S9). The results of our research and the reported data are definitely a qualitative leap, considering that so far, complete mitochondrial DNA data have not been available from the region, and Y-chromosomal data containing 23 STRs have not been reported before.

Besides revealing present-day diversity, it is of great importance to evaluate genetic continuity or transformation between present-day and ancient populations. To explore this, further medieval samples, regional genetic transects, and complete genome analyses are aimed. The follow-up project involves the study of medieval cemeteries from the same Odorheiu Secuiesc region to monitor the population history of the Székelys [82,83,84,85,86,87,88,89].

## Data Availability

The data presented in this study are openly available in the European Nucleotide Archive at [https://www.ebi.ac.uk/ena/browser/search], accession number [PRJEB52529] accessed on 20 December 2022 and at Y-STR Haplotype Reference Database (YHRD), accession number: [YA00612] (becomes available with Release 69).

## Supplementary Materials

The following supporting information can be downloaded at: https://www.mdpi.com/article/10.3390/genes14010133/s1, Figure S1: Ward Hierarchical Clustering; Figure S2: PCA plot with 56 modern and three ancient populations; Figure S3: Phylogenetic tree of mitochondrial group A12a; Figure S4: G2a median-joining network; Figure S5: Q- M242 median-joining network based on 16 Y-STRs data; Figure S6: R1a-M458 median-joining network; Figure S7: R1a -Z93 median-joining network; Figure S8: Multidimensional scaling (MDS) plot; Figure S9: Network about the local movement of the sample donors’ ancestors; Table S1: Information about the samples; Table S2: Tested Y chromosomal STR (PowerPlex23) and SNP data with haplogroup prediction; Table S3: Modern-day distribution peaks of the mtDNA sub-haplogroups found in the Székely population; Table S4: PCA frequency data and information about the populations; Table S5: Tamura Nei population pairwise FST and *p*-values; Table S6: References for Neighbour Joining phylogenetic trees; Table S7: Modern-day distribution peaks of the Y chromosomal subhaplogroups found in the Székely population; Table S8: Y chromosomal RST and p values; Table S9: 23 Y-STR data; Table S10: Heteroplasmy test for mtDNA with Mutserve; Table S11: Table of mitochondrial DNA diversity in three Székely populations based on the sequence data of the HVR-I region; Table S12: Table of population pairs FST values and significance testing.

## Abbreviations

AMOVA	Analysis of Molecular Variance
HG	Haplogroup
MDS	Multidimensional Scaling
MJ	Median-joining
NGS	Next-generation Sequencing
NJ	Neighbor-joining
PCA	Principal Component Analysis
SNP	Single Nucleotide Polymorphism
STR	Short Tandem Repeat
Y-STR	Y chromosome Short Tandem Repeat
YHRD	Y-STR Haplotype Reference Database
ybp	years before present
