# Supplementary material for: High Coverage Mitogenomes and Y-Chromosomal Typing Reveal Ancient Lineages in the Modern-Day Székely Population in Romania
Source: Genes (Basel). 2023 Jan 3;14(1):133. doi: 10.3390/genes14010133 (PMC9858685; doi:10.3390/genes14010133)
Supplement: Supplementary file 1 [file genes-14-00133-s001.zip › Supplementary_Information_Borbely-et-al.pdf]

High Coverage Mitogenomes and Y-Chromosomal Typing Reveal Ancient Lineages in the Modern Day Székely Population in Romania

Supplementary Information

Noémi Borbély, Orsolya Székely, Bea Szeifert, Dániel Gerber, István Máthé, Elek Benkő, Balázs Gusztáv Mende, Balázs Egyed, Horolma Pamjav, Anna Szécsényi-Nagy

Figure S1: Ward Hierarchical Clustering

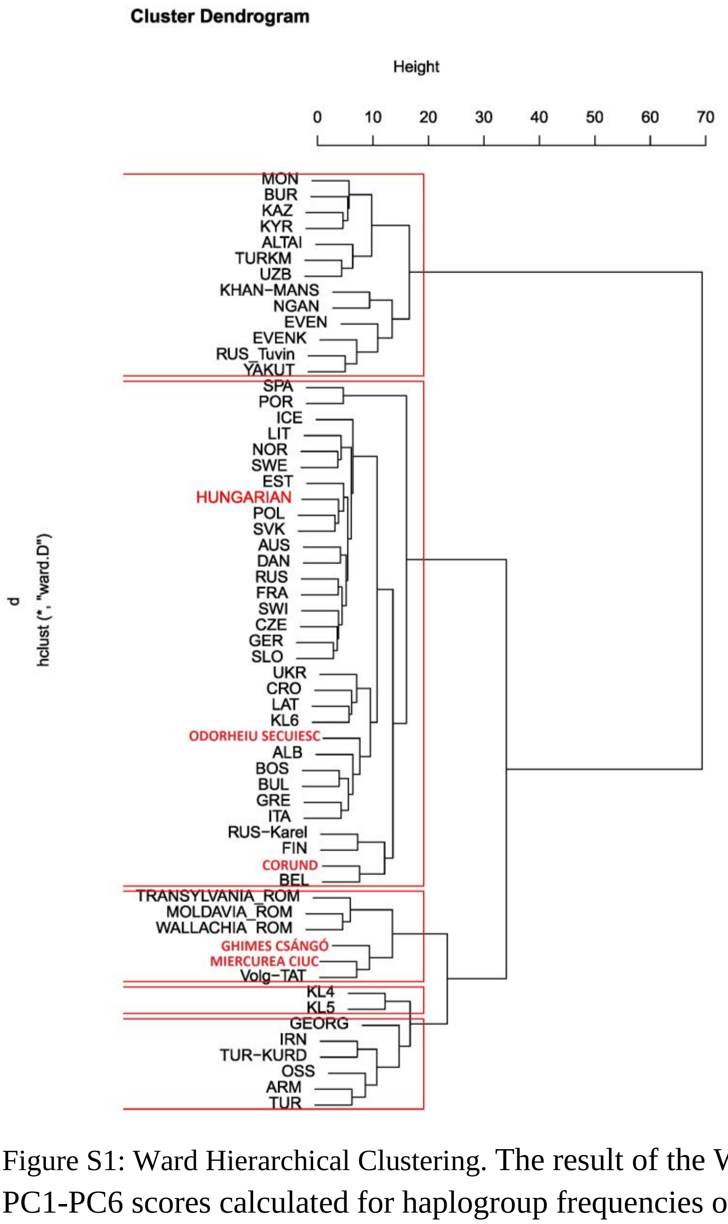

Figure S1: Ward Hierarchical Clustering. The result of the Ward cluster analysis is based on PC1-PC6 scores calculated for haplogroup frequencies of 56 modern-day and three ancient populations' (the datasets are the same as used for the PCA analysis). The results show that the investigated Székely group forms a sub branch with modern European populations: Albanian, Bosnian, Bulgarian, Greek and Italian groups are on the same branch.

Figure S2. PCA plot with 56 modern and three ancient populations

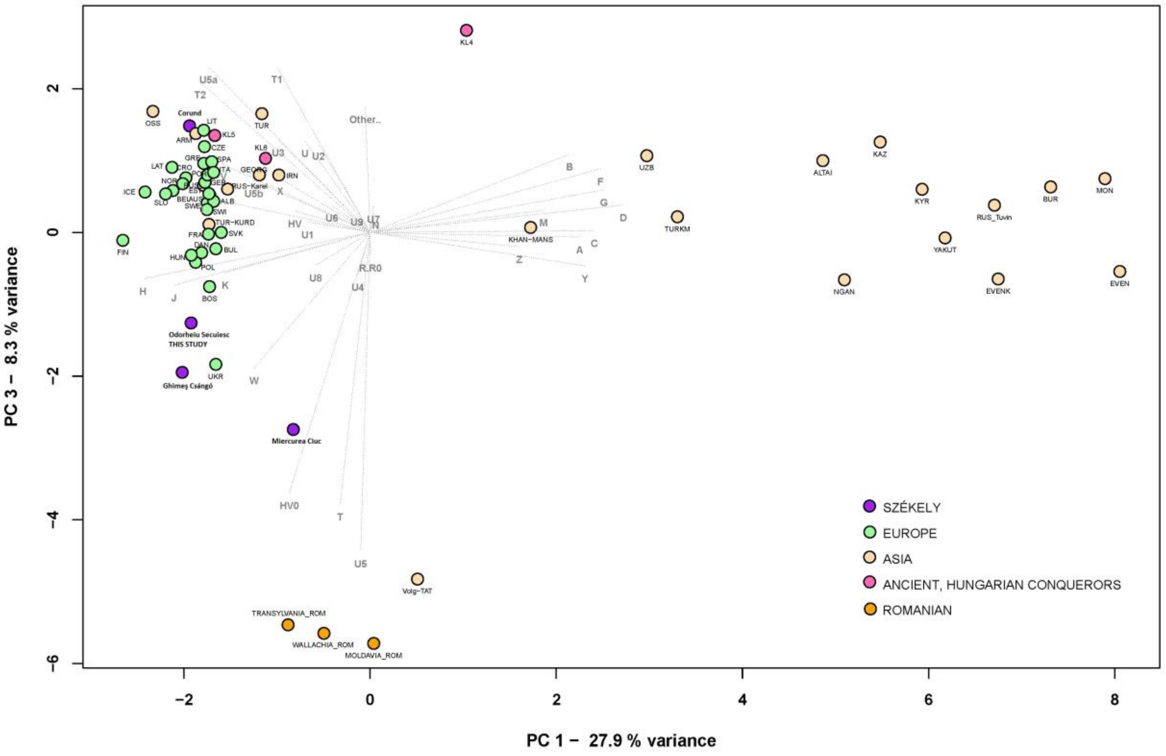

Figure S2. PCA plot with 56 modern and three ancient populations, representing first and third principal components (36.2% of the variance). PCA analysis based on haplogroup frequencies in Eurasian modern populations and three ancient populations (Hungarian conquerors, group KL4-6 based on Kovács, 2013 [82], see Supplementary Table S3) from Hungary. The investigated Székely population and previously examined Székely groups are marked in purple, the ancient populations from Hungary are indicated in pink, Romanian populations are in orange. Europeans are colored green, Asian populations have a drab color. The Székely populations which clustered together on the PC1-PC2 plot split up on the PC1-3 plot while the Romanian groups located further to the Székely groups along the PC3 axis.

Figure S3. Phylogenetic tree of mitochondrial group A12a.

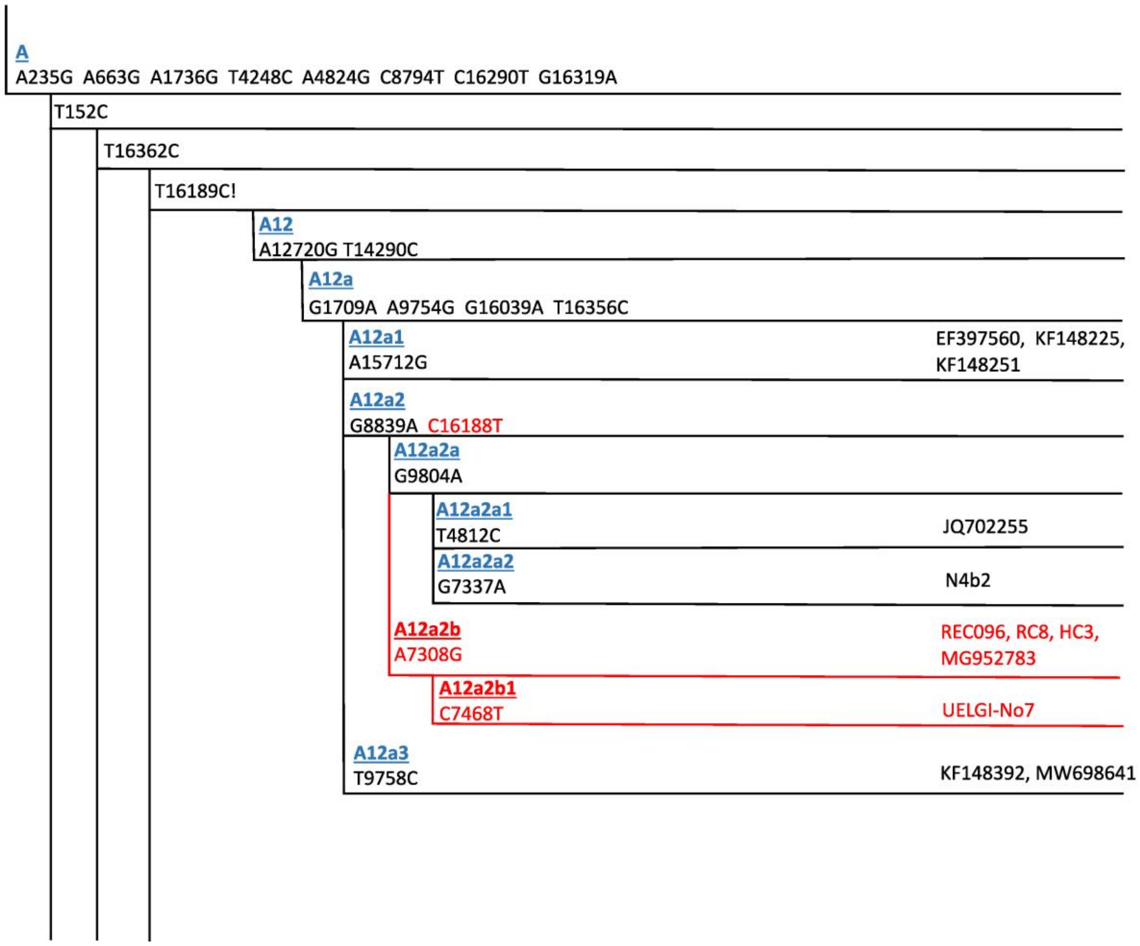

Figure S3. Phylogenetic tree of mitochondrial group A12a. Taking into consideration the currently used nomenclature of the phylogenetic tree of global human mitochondrial DNA variation we described new subbranches on the A12a tree, named as A12a2b and A12a2b1. These fit perfectly into the current nomenclature, the new subbranches do not affect the previously named samples and branches. All data were used from MTree [83], and Ian Logan mtDNA [84], the codes of the samples are indicated at the end of each line. The new branches are colored in red, the A12a2b group is made up of samples from the investigated Székely group (REC096, modern-day sample) from Hungary (MG952783: Hungarian present-day sample; HC3: mediaeval, classical Hungarian conqueror sample), and from Bolshie Tigani and Uyelgi (RC8 [52], and UELGI-No7 (see in Csáky et al, 2020 [49], under ID SB7) mediaeval Hungarian-related individuals).

Figure S4. G2a median-joining network

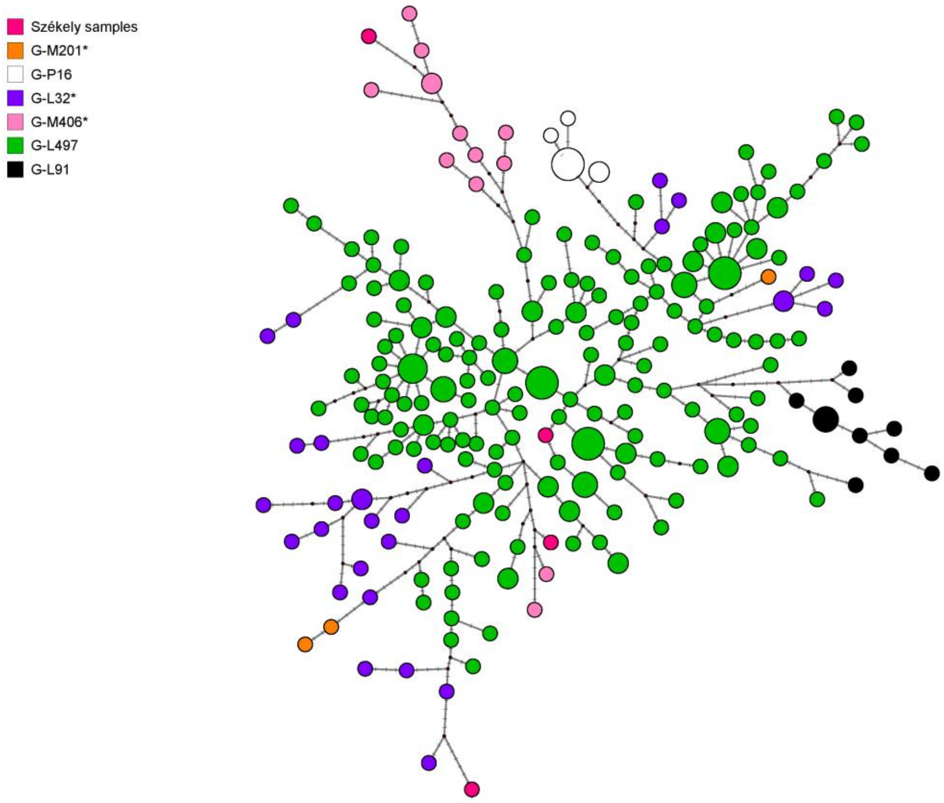

Figure S4. G2a median-joining network: The network was constructed based on 17 Y-STRs, the data used for the network are all from the Tyrolean region of Austria [67]. Circles represent distinct haplotypes, the size of the circles is proportional to the haplotype frequency (the smallest circle corresponds to one individual), the coloring corresponds to haplogroup subclades where the Székelys are highlighted.

Figure S5: Q- M242 median-joining network

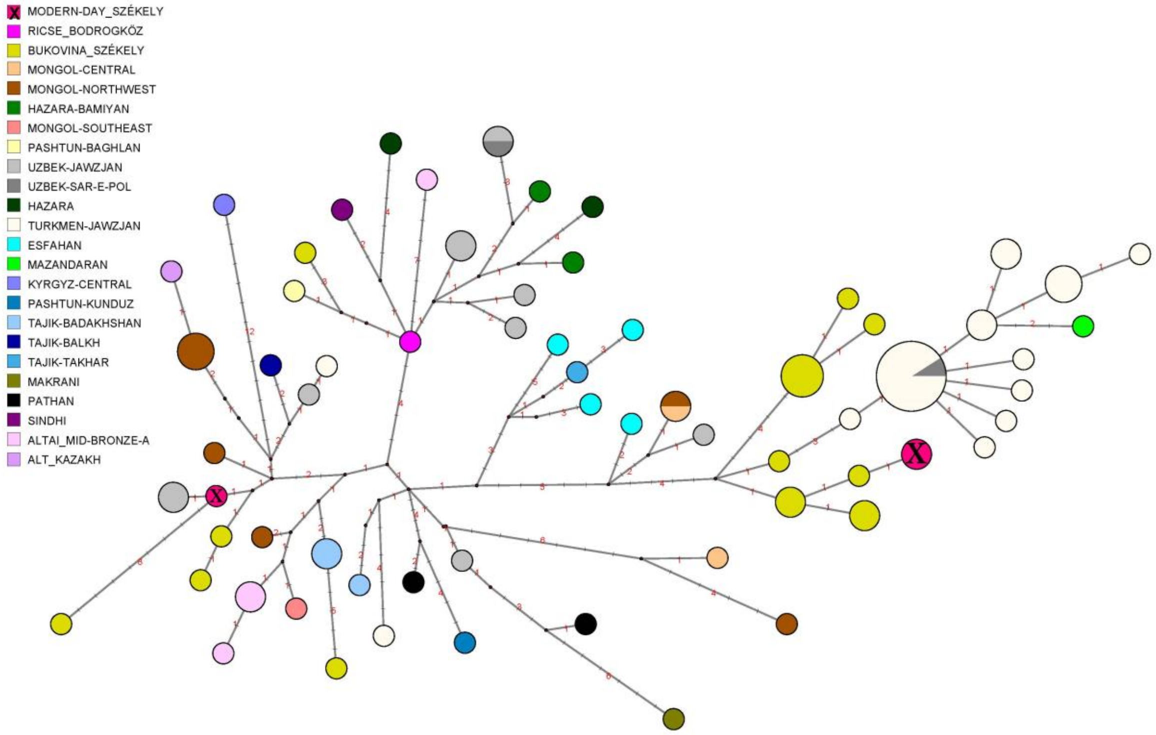

Figure S5: Q- M242 median-joining network based on 16 Y-STRs data. The median-joining network of the haplogroup Q represents populations of diverse geographic origins. The Székely Y chromosome lineages place together with Székely samples of Bukovina.

Figure S6: R1a-M458 median-joining network

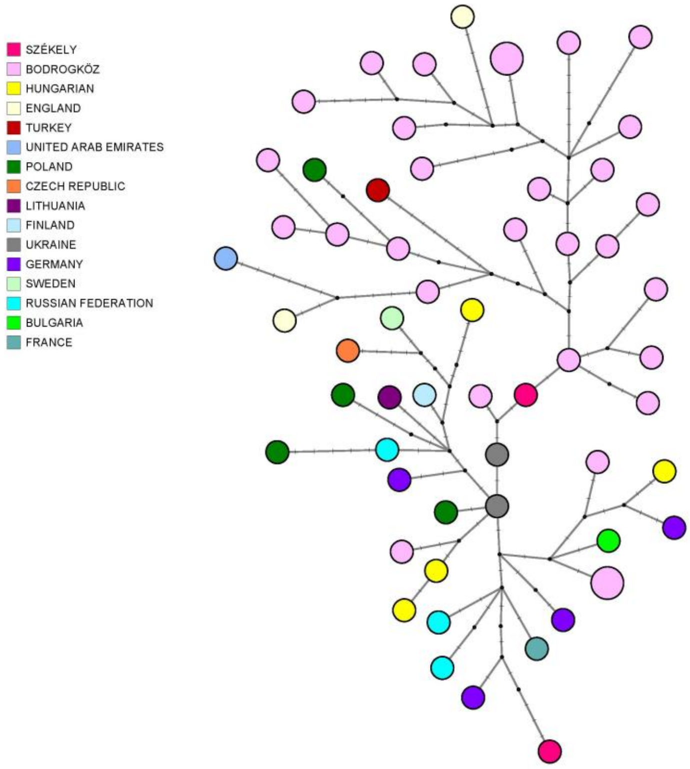

Figure S6: R1a-M458 median-joining network. The analysis was performed based on 23 STRs. We collected data from the public Family Tree Y-DNA database filtering for the subgroup R1a-M458-L1029 and used R1a-M458+ data from Pamjav et al 2017 (Hungarian samples from the Bodrogköz region of northeast Hungary) [22]. This network doesn't show geographically relevant distribution of the lineages, but shows the divergence of the R1a-M458 types within the Hungarian Bodrogköz population, and the connection of the Székely lineages to some parts of it.

Figure S7: R1a -Z93 median-joining network

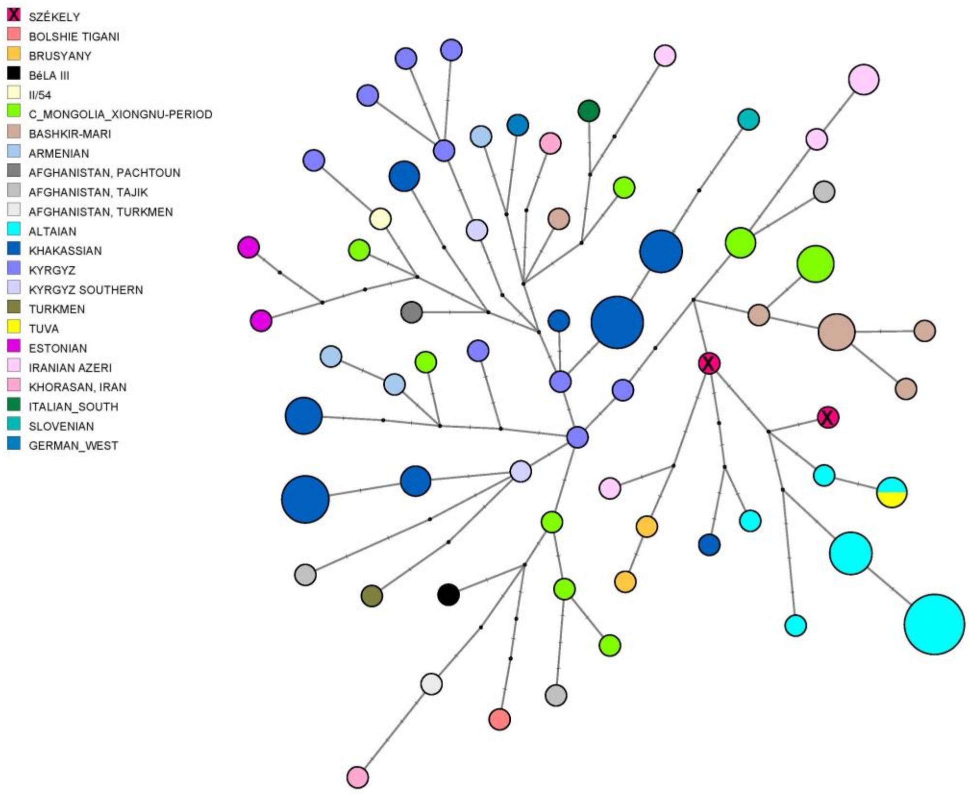

Figure S7: R1a -Z93 median-joining network based on 16 Y-STRs data. For comparison, we collected Y-STR data from the Family Tree Y-DNA database R1a page and filtered the samples for Z93, moreover, we include data from Underhill et al., Olasz et al., Dudás et al., and Szeifert et al. [85, 76, 86, 52]. Hungarian King Béla III and other skeletal remains originating from the Royal Basilica of Székesfehérvár show a great genetic distance from the Székely samples, just like the Bashkirian Mari males. Samples from Europe (Lithuania, Germany), and Bahrain on the one hand, and Syria, and Kyrgyzstan on the other hand (haplogroup Z2124 - R1a1a1b2a2a (ISOGG v15.73) [35]) are the closest to the two Székely samples respectively.

Figure S8. Multidimensional scaling (MDS) plot

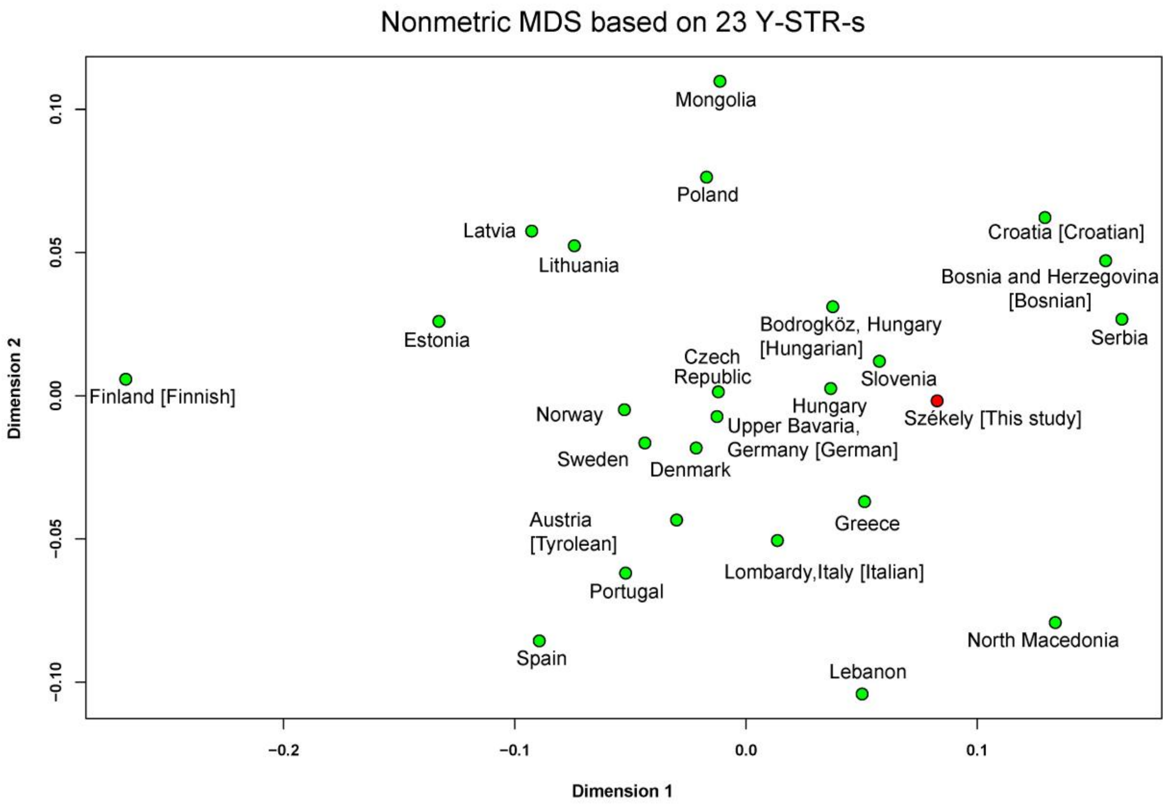

Figure S8. Multidimensional scaling (MDS) plot constructed on R_{ST} genetic distances of 23 Y-STR-based haplotype frequencies. The MDS (Kruskals' non-metric multidimensional scaling) based on 23 Y-STRs (see Supplementary Table S9) shows the strongest paternal genetic connection of the Székelys to the Slovenians (R_{ST} *p*-value is not significant), Hungarians and to Hungarians from the Bodrogköz region (North-East Hungary), and to the Greeks. The results of the MDS correspond to the clustering presented in Figure 11. The R_{ST} and *p*-values (see Supplementary Table S8) were generated on the YHRD website [87].

Figure S9. Local movements of the sample donors’ ancestors

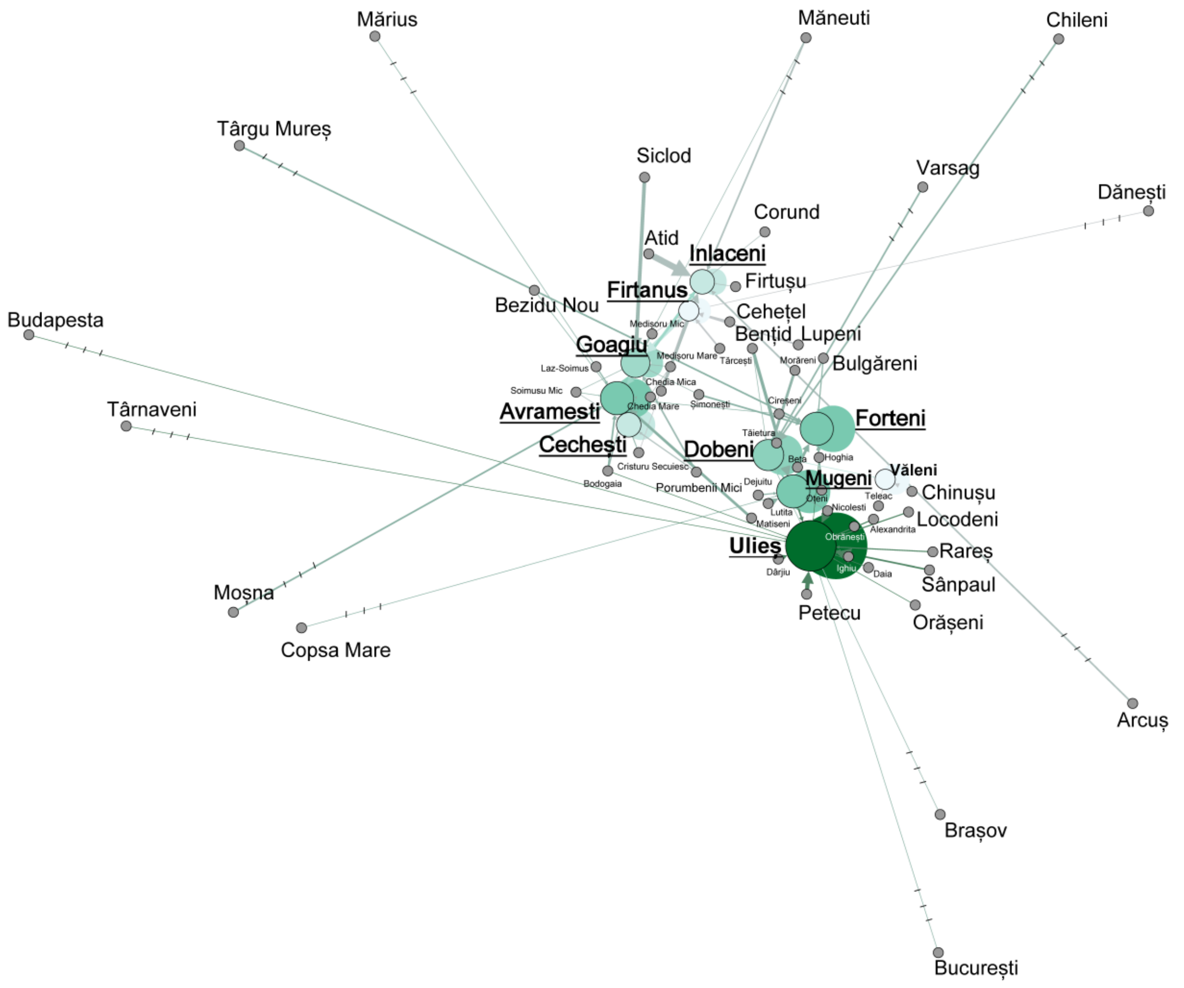

Figure S9. Network about the local movement of the sample donors’ ancestors. The birthplaces of the sample donors and their parents and grandparents are marked with circles and the generations are connected by a line. If they were not born in the same place, two circles are connected, if they were born in the same village, the circle is connected to itself. It can be seen that the villages where the sampling was carried out stand out the most, indicating that most of the sample donors' ancestors lived in the locality. The relative position of the settlements corresponds to their geographical location and is proportional to the distances (except for the more distant settlements indicated by dashed lines at the edges of the figure). The size of the circles is proportional to the number of persons from the settlement. The figure was created in Gephi software, using ‘GeoLayout’ plugin, using latitude/longitude information of the settlements [88].

Comparison of the modern-day Székely groups based on HVR-I sequences

The comparability with reference data from the Carpathian Basin and Romania is rather limited considering that the complete mitochondrial DNA data is missing from the region. We co-analysed the Odorheiu Secuiesc/Székelyudvarhely region's population also sequence-based with previous data on modern-day Székely populations from Miercurea Ciuc /Csíkszereda [17] and Corund/Korond [15]. Given that only Hyper Variable Region I (HVR-I) data are available from these previous, ethnically and geographically relevant studies, we used the HVR-I part of the mitogenomes for the comparison.

The aim of this comparison was to find out whether there is a significant genetic difference between the Székely populations living in different regions of Transylvania.

Table S11: Table of mitochondrial DNA diversity in three Székely populations based on the sequence data of the HVR-I region

	Odorheiu Secuiesc **region (This study)**	Miercurea Ciuc [17]	**Corund** [15]
n (number of sequences)	115	178	76
number of haplotypes (HVR-I region)	64	98	50
polymorphic sites	52	84	54
random match probability	2.49%	1.96%	3.25%
mismatch distribution	4.321	4.734	4.081
genetic diversity	0.982	0.984	0.974

Table of mitochondrial DNA diversity in three Székely populations based on the sequence data of the HVR-I region (np 16024-16383), excluding the length polymorphisms of the polyC strands.

The genetic diversity is very similar in the three populations, the highest in Miercurea Ciuc. The random match probability (RMP) is a rather high in each case given that we compare relatively small isolated populations [89].

Based on the results of the AMOVA analysis, 98.36% of the total variability between sequence pairs is due to differences within populations and only 1.64% of the total variance can be attributed to differences between populations.

In estimating the significance (p) of F_{ST} values between population pairs, we tested the null hypothesis that population pairs do not differ in their genetic structure. Based on the F_{ST} values, the genetic distance of the three Székely populations can be considered as significant,

but the variance between the populations given by the F_{ST} values does not exceed 2% in any of the population pairs.

Table S12: Table of population pairs FST values and significance testing.

	Corund	Miercurea Ciuc	Odorheiu Secuiesc
Corund	-	0.01695*	0.01523*
Miercurea Ciuc	*0.0000*	-	0.01806*
Odorheiu Secuiesc region	*0.0000*	*0.0000*	-

Table of population pairs F_{ST} values (above diagonal) and significance testing (p-value, below the diagonal). * = F_{ST} values observed between population pairs show a significant difference at the p = 0.05 significance level.

Based on mtDNA haplogroup distribution and genetic distance calculation, the previously investigated Székely population from Corund [15] was the most similar to the newly investigated Székely population from the Odorheiu Secuiesc region.

These comparative analyses demonstrate a moderate level of regional variability of the Székelys, the importance of studying multiple Székely regions, and also indicates that by combining the haplogroup data of the three Székely populations we obtain a dataset that can adequately represent the recent Székely population in further comparisons with other populations.
